# Does patient positioning influence blood loss and transfusion rate in hip replacement for femoral neck fractures? A single-centre, retrospective chart review

**DOI:** 10.1186/s12891-021-04375-6

**Published:** 2021-05-28

**Authors:** Thomas Haider, Jesse Seilern und Aspang, Claudia Gahleitner, Stefan Plesser, Stefan Hajdu

**Affiliations:** Department of Orthopaedics and Trauma Surgery, Medical University of Vienna, Waehringer Guertel 18-20, A-1090 Vienna, Austria

**Keywords:** Femoral neck fracture, Hip arthroplasty, Hip replacement, Blood loss, Patient positioning

## Abstract

**Background:**

We compared blood loss and transfusion frequency between the lateral decubitus and the supine position in patients undergoing hip replacement surgery due to femoral neck fractures.

**Methods:**

We retrospectively included femoral neck fracture patients treated with either hemi (HA) or total hip arthroplasty (THA). We included a total of 626 patients, of which 313 patients underwent surgery in the lateral decubitus position and 313 patients in the supine position. Preoperative and day 1 postoperative blood measures including hemoglobin (Hb), hematocrit (Hct), and red blood cell count (RBC) were evaluated, as well as transfusion records analyzed.

**Results:**

The following decrease of laboratory parameters between pre- and 1st day postoperative measures was noted: RBC: -0.77 G/L (± 0.5 G/L, median = -0.80 G/L; range: -0.50 – -1.10 G/L); Hct: -7.08 % (± 4.7 %, range: -4.70 – -9.90 G/L); Hb: -2.36 g/dL (± 1.6 g/dL, range: -1.50. – -3.40 g/dL). We did not observe significant differences in transfusion frequency between the two study cohorts (*p* = 0.735 for THA, *p* = 0.273 for HA). No influence of patient positioning on Hb-decrease, Hct-decrease, or RBC-decrease was noted in our two-way ANOVA models with consideration of implant type and fixation technique (F(3,618) = 1.838, *p* = 0.139; F(3,618) = 2.606, *p* = 0.051; F(3,618) = 1.407, *p* = 0.240).

**Conclusions:**

We did not observe  significant differences in perioperative blood values and transfusion rates in association with patient positioning in patients undergoing hip replacement surgery for femoral neck fractures.

**Level of evidence:**

Level III, retrospective cohort study.

## Introduction

Hip fractures account for a high number of adult morbidity and mortality [1]. Treatment of these patients still poses a challenge with a, over recent years unchanged, 1-year mortality rate of up to 36 % [2, 3]. Aside from surgery, these patients require intensive postoperative rehabilitation and, in some cases, even life-long extramural support due to permanent disability to varying extent. Because of demographic development, the global prevalence of patients with a disability consecutive to femoral neck fractures is predicted to further increase to 21 million patients by 2040, up from 4.5 million people as of today [3, 4]. Especially in elderly patients with displaced femoral neck fractures available literature supports treatment with prosthetic hip replacement rather than internal fixation [3, 5]. Both total hip arthroplasty (THA) and hip hemiarthroplasty (HA) are being utilized in these cases. While THA is generally used in younger, more active patients, HA is the treatment of choice in elderly patients without preexisting severe hip osteoarthritis. However, guidelines and cut-offs directing the choice of implant remain controversially discussed and are primarily guided by the treating surgeon´s experience [3, 5]. Also, a recently published large prospective randomized, controlled clinical trial found only marginal differences between both treatment options at 2-years follow-up [1].

Patients sustaining femoral neck fractures, due to demographic development, became frailer over recent years, thus resulting in increased complexity of perioperative patient care [6]. Estimates suggest frailty rates of up to 16 % of community-dwelling older adults [7–9].

Results of previous studies have demonstrated a lower blood loss in lateral decubitus positioning compared to supine positioning during surgery in elective primary THA cases [10–13]. On the contrary, a study by *Pace and Yousef* has demonstrated no effect of positioning in primary THA [14]. There is no available literature regarding this issue in hip fracture patients undergoing hip replacement surgery to our knowledge. Therefore, we aimed to close this gap and investigate whether patient positioning influenced perioperative blood loss and transfusion rates in these patients.

Our main hypotheses were that patient positioning during hip replacement surgery in femoral neck fracture cases (I) does not influence intraoperative blood loss and (II) does not lead to increased transfusion frequency.

## Materials and methods

We performed a retrospective chart review and matched pair analysis. We identified femoral neck fracture patients treated with either HA or THA at our institution between October 1997 and December 2016.

Patients eligible for inclusion were skeletally mature, at least 18 years of age, and were referred to our department for a traumatic femoral neck fracture. Pathologic fractures, periprosthetic fractures, revision surgery of any sort, or nonunion cases were excluded. Patients with multiple injuries were also excluded.

**Fig. 1:**
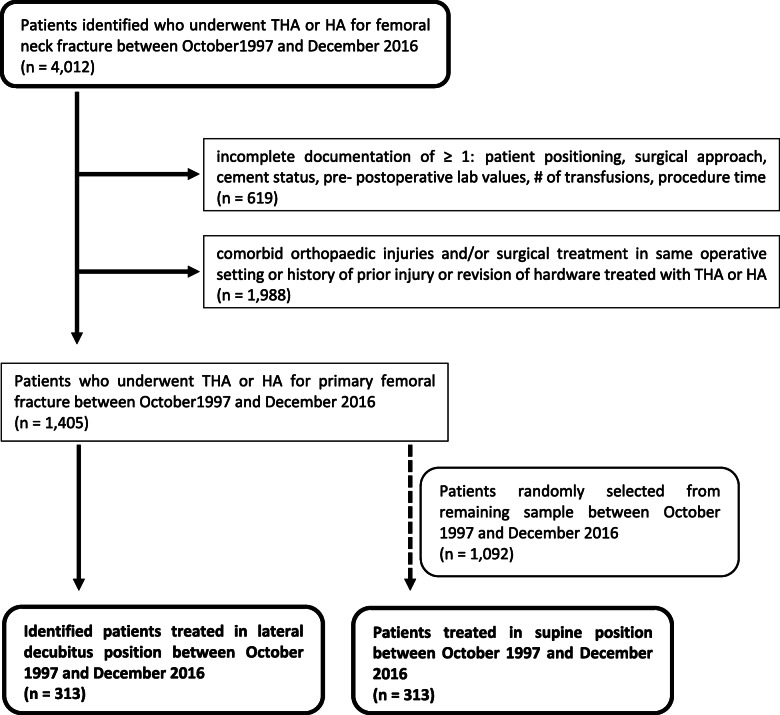
Flow diagram of the study population (THA = total hip arthroplasty, HA = hemiarthroplasty)

In a first step, we identified 4,012 femoral neck fracture patients who had received prosthetic hip replacement in the assessed timeframe. Following application of exclusion criteria, 1,405 patients were allocated to further analysis. Since supine positioning was the predominant positioning in our collective, we decided to identify patients treated in the lateral decubitus position first (*n* = 313). Consecutively, a randomly picked cohort of 313 patients from the supine positioned group comparable in age and prosthesis type (HA or THA) was assigned to the definitive study population (Fig. 1).

The study was approved by the local institute´s ethics committee of the Medical University of Vienna (protocol no. 1340/2018). Additional clinical data including age, sex, type of approach, surgery time, preoperative blood values and blood values from the 1st postoperative day (hemoglobin, Hb in g/dL; hematocrit, Hct in %, and red blood cell count, RBC in G/L), as well as transfusion records were collected and analyzed. All surgeries were performed either under general anesthesia or subarachnoid spinal block as assigned by the attending anesthesiologist. Lateral positioning involved the direct lateral approach (Hardinge/Bauer approach, *n* = 284), the anterolateral approach (Watson-Jones approach, *n* = 20), and the posterior approach (Moore approach, *n* = 9), whereas either the direct lateral (*n* = 116), the anterolateral (*n* = 189) or the direct anterior approach (modified Smith-Petersen approach, *n* = 8) to the hip was used in supine positioned cases depending on the surgeon’s preference. In all cases, the Aesculap hip implant system® (B.Braun®, Germany) was utilized. In both cohorts either uncemented or cemented femoral stem fixation was used. In all THA cases uncemented cup fixation was used. Over the 10-year observational period, a total of 34 surgeons performed surgery in included cases. Decision regarding implantation of either HA or THA was based on patient´s age, co-morbidities, and prior level of activity and ambulation. All included patients presented with Garden III- or Garden IV-type femoral neck fractures.

The transfusion protocol of our institution provides for administration of 2 concentrated red-blood-cell units if Hb-values decrease below 8.0 g/dL, which is in accordance with current international transfusion guidelines [15].

### Statistical analysis

Due to the large sample size, data were reviewed graphically and considered normally distributed. Using the Cochran–Mantel–Haenszel test we tested whether an association between patient position (THA lateral, THA supine and HA lateral, HA supine, respectively) and the need for a blood transfusion exists while simultaneously taking the usage of cement into account. To assess the influence of implant type and positioning (THA lateral, THA supine and HA lateral, HA supine, respectively), as well as fixation technique (cemented vs. uncemented) on RBC-, Hb-, and Hct-differences we utilized the two-way ANOVA. Using the Levene-test we found a violation of the assumption on homoscedasticity for Hb-difference. Comparison between the two groups were performed using the Fisher´s exact test for binary variables and the T-test for independent variables in case of continuous variables. All values are given in mean ± standard deviation (SD) if not stated otherwise. A *p*-value < 0.05 was considered statistically significant. All calculations were performed using SPSS (Version 25, IBM®, USA).

## Results

### Patient characteristics

The final study population consisted of 626 patients, of which 313 (50 %) were positioned in the lateral decubitus and 313 patients (50 %) in the supine position during surgery, respectively. Tables 1 and 2 summarize demographics and evaluated variables. Overall, the mean age was 79 years (± 11 years, median = 81 years, min.-max.: 41–99 years) and the population was predominately female with 455 females (72.7 %) vs. 171 males (27.3 %). We recorded intra-hospital mortality in 1 patient (0.16 %) who underwent surgery in the lateral decubitus position. Prosthetic replacement was predominately performed using hip hemi-arthroplasty accounting for a total of 460 patients or 230 patients in each positioning group, respectively. The remainder of the study population, a total of 166 patients or 83 patients in each positioning group, respectively, underwent total hip replacement. The majority of patients underwent prosthetic procedures using uncemented implants (400 uncemented vs. 226 cemented cases). Time to surgery was comparable between the positioning groups (lateral vs. supine, median [IQR]: 21.0 [10–31] vs. 21.5 [6–33] hours; *p* =0.082). Overall, the mean operation time was significantly lower in the lateral positioning group (87 min vs. 110 min, *p* < 0.001). A total of 11 patients (1.8 %) received preoperative red blood cell units due to preoperative anemia with 8 patients from the supine and 3 patients from the lateral positioning group.

**Table 1:**
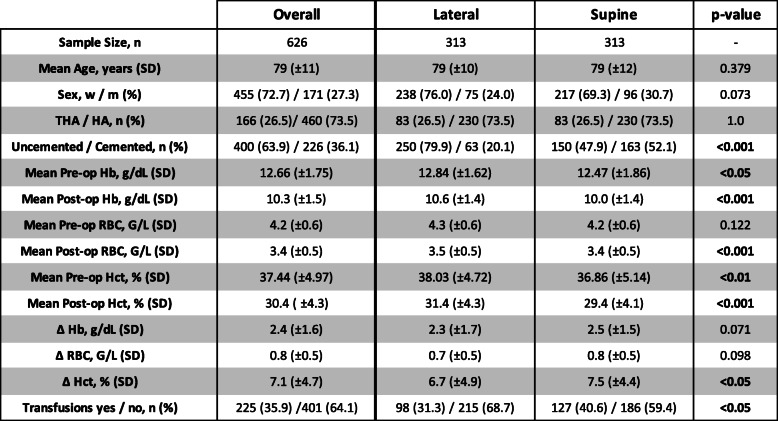
Comparison of demographics and patient characteristics between the two study groups

*SD *Standard deviation, *THA *Total hip arthroplasty, *HA *Hemi hip arthroplasty, *Hb *Hemoglobin, *RBC *Red blood count, *Hct *Hematocrit

**Table 2:**
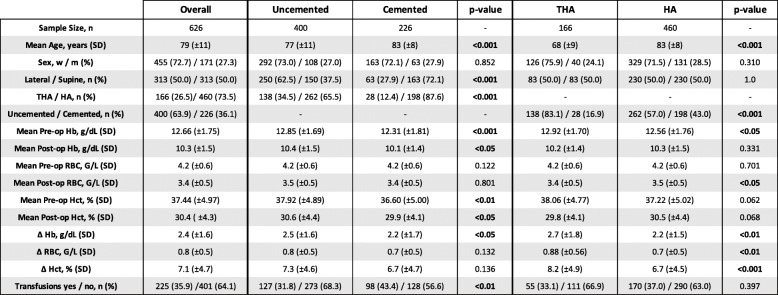
Demographics and characteristics of the patient population arranged by implant type and cement status

*SD *Standard deviation, *THA *Total hip arthroplasty, *HA *Hemi hip arthroplasty, *Hb *Hemoglobin, *RBC *Red blood count, *Hct *Hematocrit

### Comparison of perioperative blood-values

For the entire study cohort, we found the following decrease of laboratory values between pre- and 1st day postoperative measures: RBC: -0.77 G/L (± 0.5 G/L, median = -0.80 G/L; range: -0.50 – -1.10 G/L); Hct: -7.08 % (± 4.7 %, median = -7.40 %; range: -4.70 – -9.90 G/L); Hb: -2.36 g/dL (± 1.6 g/dL, median = -2.4 g/dL; range: -1.50. – -3.40 g/dL). For the two different implant types (HA vs. THA), we separately tested if there was a significant difference in transfusion frequency when taking both patient positioning (supine vs. lateral decubitus) and fixation technique (cemented vs. uncemented) into account. There was no significant difference between both implant types (*p* = 0.735 for THA, *p* = 0.273 for HA). Also, Hb-decrease, Hct-decrease, and RBC-decrease were not significantly influenced by positioning when considering implant type and fixation technique in our two-way ANOVA models (F(3,618) = 1.838, *p* = 0.139; F(3,618) = 2.606, *p* = 0.051; F(3,618) = 1.407, *p* = 0.240).

## Discussion

In the present study, we found that perioperative blood loss and transfusion rates were not associated with patient positioning during hip replacement surgery for femoral neck fractures. To our knowledge, this was the first study to investigate the influence of patient positioning on blood laboratory parameters in femoral neck fracture patients undergoing hip replacement surgery.

There is, however,  literature available on this topic involving primary THA cases. Nonetheless, there is an ongoing debate whether and how patient positioning influences blood loss in hip replacement surgery. Several studies have demonstrated decreased blood loss following surgery in the lateral decubitus position in comparison to supine positioning [10–13]. Contradicting these results, *Pace and Yousef* reported no difference in blood loss between supine and lateral patient positioning in a prospective, randomized clinical trial [14]. Our study findings agree with the latter report and translate these findings to hip fracture cases.

There are several significant differences in patient characteristics to be considered when comparing elective primary total hip replacement for osteoarthritis and prosthetic replacement for hip fractures, with a higher rate of co-morbidities, as well as increased morbidity and mortality in the latter group [16]. Preoperatively, many hip fracture patients are at high risk for postoperative anemia, if not already present at admission. There is a clear association between anemia and morbidity and mortality [17, 18]. Also, in elective THA cases anemia was associated with increased risk of periprosthetic joint infections with assumedly comparable effects in hip fracture patients [19]. While anemia was not associated with mortality rates after elective primary THA, it was strongly associated to morbidity and mortality in hip fracture patients [17, 19]. These findings have urged treating physicians to reduce blood loss and prevent anemia, and treat anemia early with transfusion of red blood cell units if necessary [17].

A near 10-fold increase in patients living with disabilities after a femoral neck fractures is expected by 2050 [3, 4]. Demographic development with an increase of the aged population and ageing baby-boomers are indicative of the expected increase of femoral neck fracture patients. Also, the number of frail patients with high morbidity and mortality rates following hip fractures is expected to rise substantially [4, 20]. We found our study population demographics to be representative and comparable to other studies involving hip fracture patients with a mean age of 79 years and predominantly female, that is  73 % of the included patient collective [1, 16, 17, 21].

 Among our study population, patients underwent different surgical approaches to the hip, which might have interfered with our results. However, a recent meta-analysis by *Wang and colleagues* reported no differences in transfusion rates comparing the direct anterior with the direct lateral approach to the hip in elective primary THA [22]. However, they did show a minor, but significant difference in blood loss favoring the direct anterior approach [22]. We only had a small group of patients (*n* = 17) who did not undergo the lateral or anterolateral approach. Therefore, we propose that the different approaches used in the presented study population did not cause a relevant bias. In total, 34 surgeons over a 10-year observation period performed surgeries of the included cases. Our department is a teaching hospital, which explains the high number of different surgeons as well as the comparatively long operation time. The duration of the procedure was significantly longer in the supine group. This is explained by the higher proportion of teaching surgeries in this patient cohort compared to the lateral group. We did not find any correlation between operation time and assessed laboratory parameters (data not shown). Therefore, we argue that this difference in operation time can be neglected when interpreting the presented results. Time to surgery might represent a confounding factor for intra- and perioperative blood loss. While timing of surgery did influence blood loss in a recently published study reporting on intertrochanteric fractures, this correlation was not found in femoral neck fracture patients [23, 24]. This discrepancy might be a result of the hip capsule limiting preoperative blood loss in femoral neck fractures. Time to surgery did not differ between the two patient cohorts in the present study, thus rendering a potential confounder in this regard unlikely in our opinion.

In our statistical model, we did not observe any differences in blood loss and transfusion rates between patients receiving HA and patients who underwent THA. However, we found a significant influence of fixation technique. Cemented stem fixation was associated with both lower blood loss and transfusion rates compared to uncemented stem fixation. This topic remains controversial, since there are conflicting reports in literature as to whether cemented stem fixation is associated with differences in blood loss compared to press-fit stem implantation [25–27]. Irrespective of surgical technique, there is clear evidence that the greater part of total blood loss in hip fracture patients arises from the fracture and the soft-tissue trauma itself rather than the surgical intervention, although blood loss seems to be pronounced in extra- compared to intracapsular fractures [27–30]. Therefore, we decided to only compare preoperative and postoperative day 1 values rather than include measures from subsequent time points, since we suggest that this comparison most likely represents possible influence by intraoperative positioning. *Foss et al.* showed that Hb-concentrations begin to increase again as early as 1 h after surgery in elective primary THA cases, which  further supports this suggestion [10].

Laboratory results in the present study were comparable to available literature. For example, *Foss et al.* reported similar mean pre- and postoperative Hb values with 12.6 g/dL and 10.3 g/dL, respectively [31]. Other studies reported comparable laboratory values further corroborating the validity of our findings [14, 27].

There is an ongoing discussion as to whether THA or HA should be performed in acute hip fracture cases [1, 32]. We did not observe any difference in our outcome measures between these two subgroups. However, comparisons between these 2 subgroups need to be considered with caution because of considerable differences in sample size and demographics.

Since this study was of retrospective design, we were not able to collect data regarding the actual and “hidden” blood loss, as this would require weighing of wound dressings, drainage volume, intraoperative amount of rinse, and fluid collection within the surgical aspirator [31]. Still, transfusion administration is solely guided by 3 variables: patient presentation, Hb concentration, and postoperative Hct drop [14]. The latter two were evaluated in the present study. Therefore, we are confident that our results are of value as the evaluated and presented measures form the cornerstone of clinical decision making.

This study has some limitations. Aside from its aforementioned shortcomings, the retrospective character is the most important limitation on its own. Based on available patient records we were not able to collect sufficient information on preexisting co-morbidities and medication of included patients. Especially anticoagulant treatment could have interfered with our results.

Taken together, we did not observe a positioning-caused difference in blood loss and transfusion rates in patients undergoing prosthetic replacement following femoral neck fractures. Our results corroborate available literature that advises to position the patient according to surgeon’s preferences and institutional infrastructure.

## Data Availability

Data will be made available upon request to the corresponding author (T.H.).
